# Activation of hypoxia-inducible factor 1 attenuates periapical inflammation and bone loss

**DOI:** 10.1038/s41368-018-0015-0

**Published:** 2018-04-13

**Authors:** Kimito Hirai, Hisako Furusho, Kiichi Hirota, Hajime Sasaki

**Affiliations:** 1 Department of Cariology, Restorative Sciences & Endodontics, University of Michigan School of Dentistry, Ann Arbor, MI USA; 2 Department of Immunology and Infectious Diseases, The Forsyth Institute, Cambridge, MA USA; 3 Department of Oral and Maxillofacial Pathobiology, Hiroshima University, Hiroshima, Hiroshima Japan; 4 Department of Human Stress Response Science, Institute of Biomedical Science, Kansai Medical University, Hirakata, Osaka Japan

## Abstract

Hypoxia (low oxygen level) is an important feature during infections and affects the host defence mechanisms. The host has evolved specific responses to address hypoxia, which are strongly dependent on the activation of hypoxia-inducible factor 1 (HIF-1). Hypoxia interferes degradation of HIF-1 alpha subunit (HIF-1α), leading to stabilisation of HIF-1α, heterodimerization with HIF-1 beta subunit (HIF-1β) and subsequent activation of HIF-1 pathway. Apical periodontitis (periapical lesion) is a consequence of endodontic infection and ultimately results in destruction of tooth-supporting tissue, including alveolar bone. Thus far, the role of HIF-1 in periapical lesions has not been systematically examined. In the present study, we determined the role of HIF-1 in a well-characterised mouse periapical lesion model using two HIF-1α-activating strategies, dimethyloxalylglycine (DMOG) and adenovirus-induced constitutively active HIF-1α (CA-HIF1A). Both DMOG and CA-HIF1A attenuated periapical inflammation and tissue destruction. The attenuation in vivo was associated with downregulation of nuclear factor-κappa B (NF-κB) and osteoclastic gene expressions. These two agents also suppressed NF-κB activation and subsequent production of proinflammatory cytokines by macrophages. Furthermore, activation of HIF-1α by DMOG specifically suppressed lipopolysaccharide-stimulated macrophage differentiation into M1 cells, increasing the ratio of M2 macrophages against M1 cells. Taken together, our data indicated that activation of HIF-1 plays a protective role in the development of apical periodontitis via downregulation of NF-κB, proinflammatory cytokines, M1 macrophages and osteoclastogenesis.

## A breath of life for root lesions

Targeting a protein complex involved in the body’s response to low internal oxygen concentrations could help treat destructive tooth root inflammations. Hajime Sasaki from the University of Michigan and colleagues in the U.S. and Japan investigated the role of the protein complex HIF-1 in oral inflammation. They induced lesions in the root area of molar teeth in mice, exposing them to oral bacteria, which led to inflammation. They injected the mice with one of two agents that activate the HIF-1 pathway. Inflammation and bone destruction in the lesions were both reduced. Activation of the HIF-1 pathway turned off another pathway, called NF-κB, involved in the immune response to infection. It also turned off genes involved in bone destruction. The results indicate that HIF-1 has a protective effect on tooth root lesions and could play a role in their treatment pending further investigations.

## Introduction

Apical periodontitis (periapical lesion) is an inflammatory and immune response caused by anaerobic polymicrobial infection of the dental pulp and root canals.^1,2^ This inflammatory condition damages tissues and characteristically results in the formation of granulation tissue and loss of the alveolar bone surrounding the dental root apex. Currently, a detailed understanding of the host factors regulating the outcome of periapical lesion is incomplete.

Oxygen is an important factor affecting acute and chronic inflammation.^3,4^ Hypoxia (low oxygen level) is a key feature of inflammatory tissues due to elevated oxygen consumption by infiltrated myeloid cells. Thus, myeloid cells must adapt to hypoxic environments and maintain the function of the innate immune system against infectious microorganisms.^3,4^ In the field of endodontics, hypoxia plays pathogenic roles in the development of periapical lesions, which involve hypoxia-inducible factor 1 (HIF-1) and potentially neutrophil-derived vascular endothelial growth factor (VEGF)-C/-D/VEGF receptor (VEGFR)-2/-3 signalling.^5–9^ However, a study examining gene expression profiles after root canal treatment in a rat periapical lesion model reported a potential role of HIF-1α in the process of periapical wound healing.^10^ Activation of the HIF-1 pathway accelerates bone regeneration, reduces inflammatory cell infiltration and promotes wound healing.^11–13^ These findings suggest that HIF-1 mediates pro-healing responses.

HIF-1 is a key transcription factor of oxygen homeostasis and plays an essential role in responses to hypoxia to recover oxygen supply at the molecular to systemic levels.^14,15^ HIF-1 is a heterodimer and consists of the HIF-1 alpha and beta subunits. Under normal oxygen level (normoxia), HIF-1α is post-translationally hydroxylated by the prolyl hydroxylase domain enzymes (PHD) and subsequently undergoes proteasomal degradation.^16,17^ Hypoxia interferes with PHD-mediated degradation of HIF-1α, leading to stabilization of HIF-1α, heterodimerization with HIF-1β and subsequent activation of the HIF-1 pathway.^17^ Activation of HIF-1 plays multifaceted roles in infection-elicited inflammation. HIF-1 regulates the bactericidal capacity of phagocytes by induction of nitric oxide.^18^ HIF-1 also regulates the functional phenotype of macrophages (M1 and M2) by modulating inducible nitric oxide (NO) synthase gene expression.^19^ Furthermore, HIF-1 is involved in the regulation of bone processes, including its development, physiological remodelling, pathogenic destruction and healing.^20^ Thus, HIF-1 plays a regulatory role in the process of bone-destructive diseases such as rheumatoid arthritis.^21^ However, the role of HIF-1 in oral inflammation including periodontitis and apical periodontitis has not been systematically investigated.

In this study, we investigated whether HIF-1 activation alters the outcome of periapical lesions in mice using two HIF-1 activating approaches, including pharmacological inhibition of PHD with dimethyloxalylglycine (DMOG) and adenovirus-induced constitutively active HIF-1α (CA-HIF1A).^22,23^ We found that both DMOG and CA-HIF1A attenuated periapical inflammation via inhibition of nuclear factor-κappa B (NF-κB) activation, subsequent inflammatory response and osteoclastogenesis.

## Results

### DMOG attenuated development of periapical lesions

**Fig. 1:**
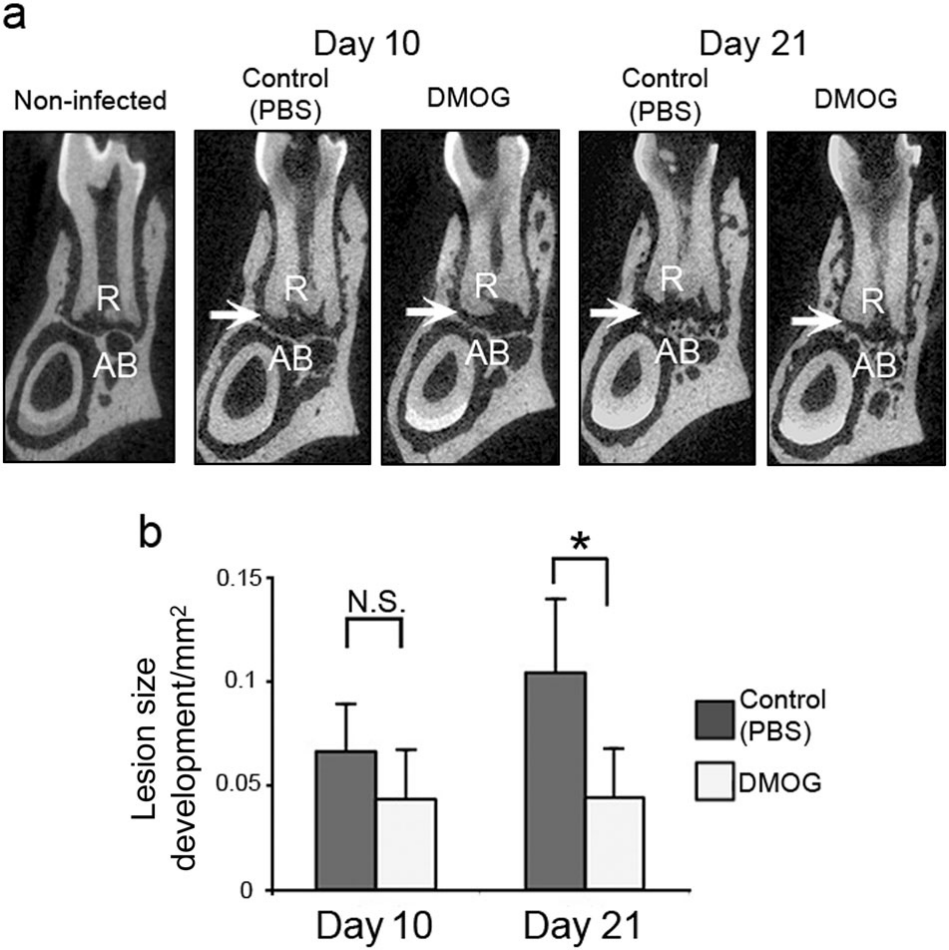
DMOG suppressed progression of periapical bone loss. **a** Representative μCT images of periapical lesions in the anterior–posterior direction. The sample number was five in each group, based on preliminary sample size calculation using G*Power 3 (Universität Düsseldorf) and our previous data sets. The position of each CT slice was the most central part of the mandibular first molar distal root (R). Arrow points to the area of the periapical lesion surrounded by radiopaque alveolar bone (AB). Non-infected: baseline control, Control: disease control group received PBS, DMOG: DMOG-treated group. **b** Size of periapical lesions development. The effect of DMOG and observation period was determined by two-way ANOVA with Bonferroni post hoc test. *P < 0.05 vs. disease control, N.S.: not significant, vertical bar: standard deviation. DMOG dimethyloxalylglycine, PBS Phosphate-buffered saline

We examined the effect of DMOG on the development of periapical lesions. As shown in Fig. 1a, all mice subjected to pulp exposure showed increased periapical radiolucency by day 21 post exposure compared with non-exposed controls, indicating pulp exposure could induce periapical lesions within 21 days. Phosphate-buffered saline (PBS)-injected disease control mice exhibited a trend of progressively increasing periapical lesion size over the observation periods. The extent of periapical lesions in the disease controls increased 56% on day 21 compared with that in day 10 (* P* = 0.11). In contrast, the lesion size in DMOG-treated mice did not change between days 10 and 21 (Fig. 1b). The DMOG treatment resulted in 57% suppression of lesion size vs. the disease control group on day 21, with statistically significant results (Fig. 1b).

### DMOG reduced inflammatory cell infiltration into periapical lesions

**Fig. 2:**
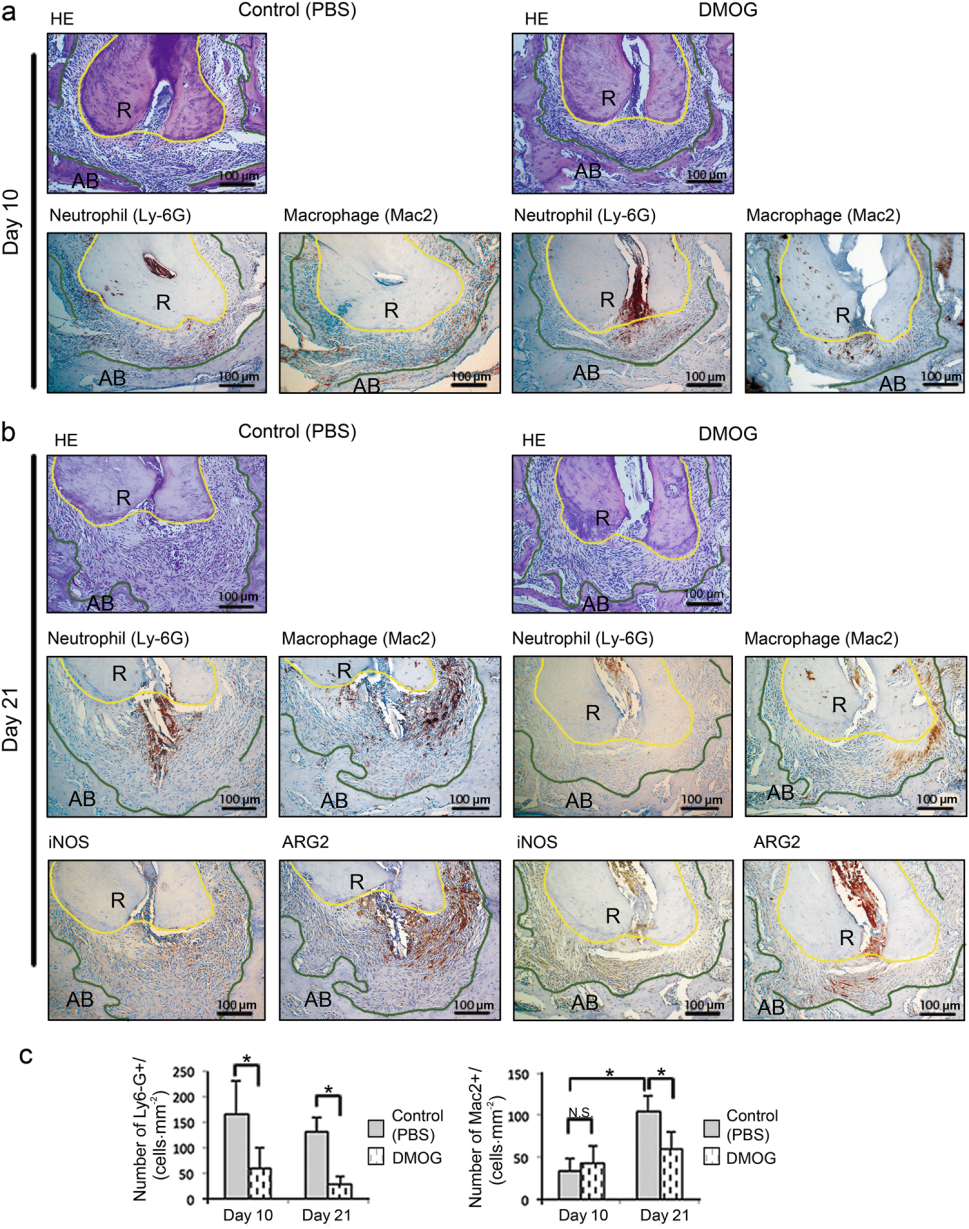
DMOG treatment attenuated inflammatory cell infiltration into periapical lesions. **a** Histology of periapical lesions on day 10 after pulp exposure. **b** Histology of periapical lesions on  day 21 after pulp exposure. In each panel, representative images of H&E staining (HE) and immunohistochemistry for neutrophils (Ly-6G), macrophages (Mac2), iNOS and ARG1 were shown. The original magnification was ×100. Polygonal yellow and green lines outline dental root (R) and the margin of residual alveolar bone (AB), respectively. The area lying between these lines is periapical lesion. Control: disease control group, DMOG: DMOG-treated group. **c** The level of myeloid cell infiltration. The effect of DMOG and observation periods on the number of neutrophils (Ly-6G) and macrophages (Mac2) per unit lesion area was determined by two-way ANOVA with Bonferroni post hoc test. *P < 0.05 vs. disease control, N.S., not significant; vertical bar: standard deviation, *n* = 5 per group. DMOG dimethyloxalylglycine, PBS Phosphate-buffered saline

The level of inflammation was histologically assessed in the same samples used in the micro-computed tomography (μCT) analysis. In disease control mice, mild to moderate levels of inflammatory cells infiltrated into granulation tissues occupying the periapical area on day 10 post-pulp exposure (Fig. 2a). On  day 21, periapical lesions further extended, and moderate levels of inflammatory cells were distributed in granulation tissues. In contrast, inflammatory cell infiltration in DMOG-treated mice was consistently mild compared with that in disease controls over the observation periods (Fig. 2a, b). Immunohistochemistry revealed that widespread infiltration of inflammatory cells in controls mainly consisted of Ly-6G(+) neutrophils and Mac2(+) macrophages on  day 21 (Fig. 2b). However, neutrophil infiltration in DMOG-treated mice was notably weak vs. that of disease controls on  day 21 (Fig. 2b). As shown in Fig. 2c, a treatment effect on the number of Ly-6G(+) neutrophils was observed over the observation periods ( *P*< 0.05 vs. controls in both days 10 and 21). The treatment effect on Mac2(+) macrophages was observed only on day 21. An observation period effect, which is a significant elevation in the number of Mac2(+) macrophages, was observed in the disease controls on  day 21 (*P *< 0.01 vs.  day 10). However, no observation period effect was observed in DMOG-treated mice. Infiltrated cells in the disease controls were a mixed population of iNOS(+) and ARG1(+) cells. By contrast, DMOG-treated mice exhibited an ARG1(+) cell-polarised profile, and iNOS(+) cells were very faint. In addition, the area of ARG1(+) cell infiltration was quite limited compared to that of disease controls.

### DMOG suppressed gene expressions related to inflammation and osteoclastogenesis in periapical lesions

We next examined the impact of DMOG on periapical gene expression profiles by quantitative reverse transcription-PCR (RT-PCR). As shown in Table 1, DMOG significantly downregulated gene expression of the NF-κB family (*Rela*, *Relb*), proinflammatory/bone-destructive cytokines (*Il1a, Tnf*) and osteoclastogenesis (*Acp5 (TRAP), Ctsk*) compared to that of disease control mice on  day 10 post-pulp exposure. Osteogenic genes (*Runx2, Sp7*) were also downregulated by DMOG at the same time. DMOG further suppressed the expression levels of the *Tnf, Acp5* and *Ctsk* genes through Day 21. *Chil3 (Ym1)*, a M2 macrophage marker was significantly upregulated on day 21.

**Table 1:** Real-time RT-PCR result of DMOG treatment

	Day 10			Day 21		
Gene	Ratio	CI	* P*-Value	Ratio	CI	* P*-Value
*Il1a*	0.6	(0.47−0.76)	0.04	1.13	(0.46−1.74)	N.S.
*Tnf*	0.59	(0.51−0.67)	0.01	0.55	(0.4–0.75)	0.02
*Nfkb1*	0.82	(0.64−1.04)	N.S.	1.19	(0.5–1.43)	N.S.
*Nfkb2*	0.82	(0.65−1.04)	N.S.	0.95	(0.65−1.39)	N.S.
*Rela*	0.68	(0.55−0.84)	0.04	1.03	(0.35−2.64)	N.S.
*Relb*	0.02	(0.02–0.03)	0.001	0.98	(0.65−1.46)	N.S.
*Nos2*	0.86	(0.73−1.01)	N.S.	1.25	(1.05−1.47)	N.S.
*Arg1*	0.54	(0.31−0.96)	N.S.	1.48	(0.91−2.41)	N.S.
*Chil3*	1.37	(0.31−1.69)	N.S.	2.93	(1.56−4.48)	0.006
*Retnla*	1.06	(0.64−1.38)	N.S.	1.19	(0.98–1.43)	N.S.
*Acp5*	0.67	(0.57−0.79)	0.02	0.35	(0.18–0.69)	0.05
*Ctsk*	0.71	(0.64−0.8)	0.04	0.33	(0.17–0.64)	0.03
*Runx2*	0.63	(0.56−0.71)	0.01	1.08	(0.54−1.58)	N.S.
*Osx*	0.64	(0.54−0.77)	0.04	0.98	(0.65−1.44)	N.S.
*Atf4*	0.98	(0.76−1.01)	N.S.	1.62	(0.95−2.77)	N.S.
*Hif1a*	0.87	(0.87−1.1)	N.S.	1.10	(0.57−1.43)	N.S.
*Car9*	0.87	(0.54−1.39)	N.S.	0.74	(0.51−1.08)	N.S.
*Vegfa*	1.24	(0.87−1.43)	N.S.	1.66	(0.87−3.18)	N.S.

Gene expression data were normalized to the internal reference gene (GAPDH) and analyzed by the 2^-ΔΔCT^ method. Ratio: the ratio of target gene expression in DMOG-treated animals vs. disease controls. The effect of treatment was determined by Student’s *t-*test

*CI 95%* confidence interval; *N.S.* not significant (*P *≥ 0.05)

### DMOG suppressed proinflammatory responses in macrophages in vitro

**Fig. 3:**
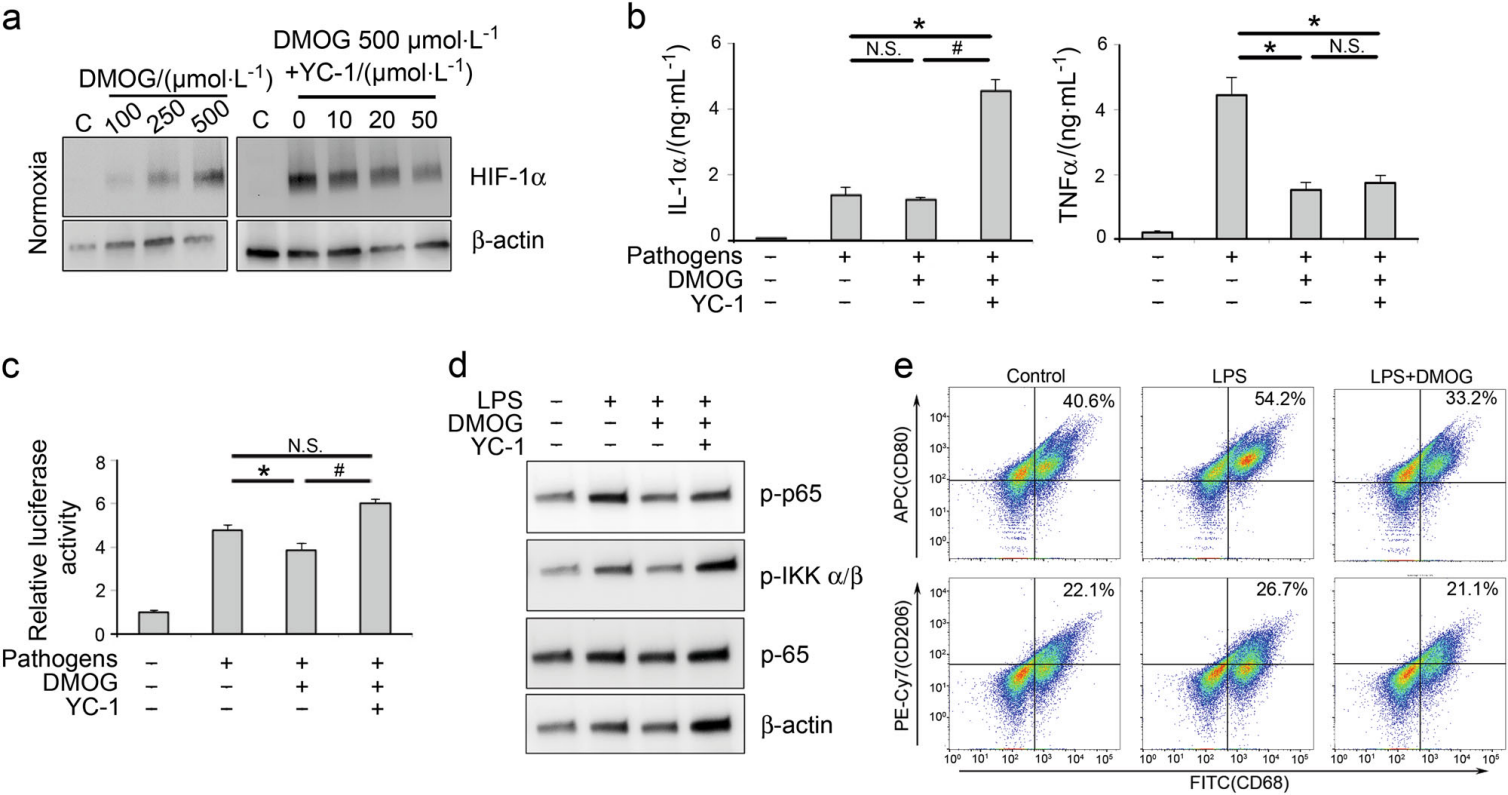
DMOG suppressed proinflammatory response in macrophages in vitro. **a** Effect of DMOG on HIF-1α protein expression (western blot). C, non-treated control. **b** Effect of DMOG on endodontic pathogen-stimulated IL-1α and TNFα. The effect of treatments on each cytokine was determined by one-way ANOVA with Bonferroni post hoc test. * P < 0.05 vs. non-treated/stimulated group, ^#^* P* < 0.05 vs. DMOG-treated/stimulated group. N.S., not significant, vertical bar: standard deviation, *n* = 3 per condition. **c** Effect of DMOG on NF-κB transcriptional activity (luciferase reporter assay). The effect of treatments was determined by one-way ANOVA with Bonferroni post hoc test. * P < 0.05 vs. non-treated/stimulated group, ^#^* P* < 0.05 vs. DMOG-treated/stimulated group. N.S., not significant, vertical bar: standard deviation, *n* = 3. **d** Effect of DMOG on NF-κB activation pathway (western blot). p-p65 phosphorylated p65, p-IKK α/β phosphorylated IKK α/β, p65, total p65. **e** Flow cytometric analysis of M1 and M2 macrophages. *x* axis: FITC fluorescence intensity, *y* axis: APC or PE-Cy7 fluorescence intensity. DMOG dimethyloxalylglycine, LPS lipopolysaccharide, YC-1 3-(5'-Hydroxymethyl-2'-furyl)-1-benzyl indazole

As macrophages are the most prominent cell type in the development of periapical lesions,^24^ the effect of DMOG on macrophage responses was assessed using mouse peritoneal macrophages. 3-(5'-Hydroxymethyl-2'-furyl)-1-benzyl indazole (YC-1) was employed to determine whether the effect of DMOG was dependent on HIF. As shown in Fig. 3a, DMOG stabilized HIF-1α protein under normoxia in a dose-dependent manner. However, co-treatment of DMOG with YC-1 resulted in downregulation of HIF-1α protein in a dose-dependent manner. The effect of DMOG and YC-1 on endodontic pathogen-stimulated cytokines was unexpectedly inconsistent. As shown in Fig. 3b, DMOG significantly suppressed tumor necrosis factor alpha (TNFα) by 31.8% compared to the positive control (stimulated/non-treated), whereas interlektin (IL)-1α production was not affected. YC-1 strongly increased IL-1α production compared to that of the positive control (>2.5-fold). However, YC-1 did not alter TNFα production compared to that of the positive control. Fig. 3c indicates the effect of DMOG on the activation of NF-κB determined by a reporter assay targeting the NF-κB response element. Although NF-κB promoter activity was significantly elevated by endodontic pathogens in 6 h, DMOG significantly inhibited the elevation of pathogen-stimulated NF-κB promoter activity. In contrast, co-treatment of DMOG and YC-1 resulted in a recovery of the NF-κB promoter activity. Furthermore, we examined whether DMOG alters phosphorylation in NF-κB activation by western blot analysis. As shown in Fig. 3d, DMOG suppressed lipopolysaccharide (LPS)-mediated phosphorylation of p65 and inhibitor of NF-κB kinase (IKK) at 8 h. As, total p65 expression was not altered by DMOG (Fig. 3d), DMOG may specifically suppress phosphorylation of p65 during proinflammatory NF-κB activation. In addition, we investigated whether DMOG alters the profile of macrophage differentiation (M1/M2 subsets in total macrophages) under LPS stimulation (Fig. 3e). Although the number of M2 macrophages (CD68+/CD206+) was not altered by DMOG, DMOG specifically inhibited LPS-stimulated macrophage differentiation into M1 cells (CD68+/CD80+) by approximately 20%. The DMOG treatment increased the ratio of M2 macrophages to M1 macrophages in vitro.

### Induction of CA-HIF1A suppressed macrophage inflammatory response in vitro

**Fig. 4:**
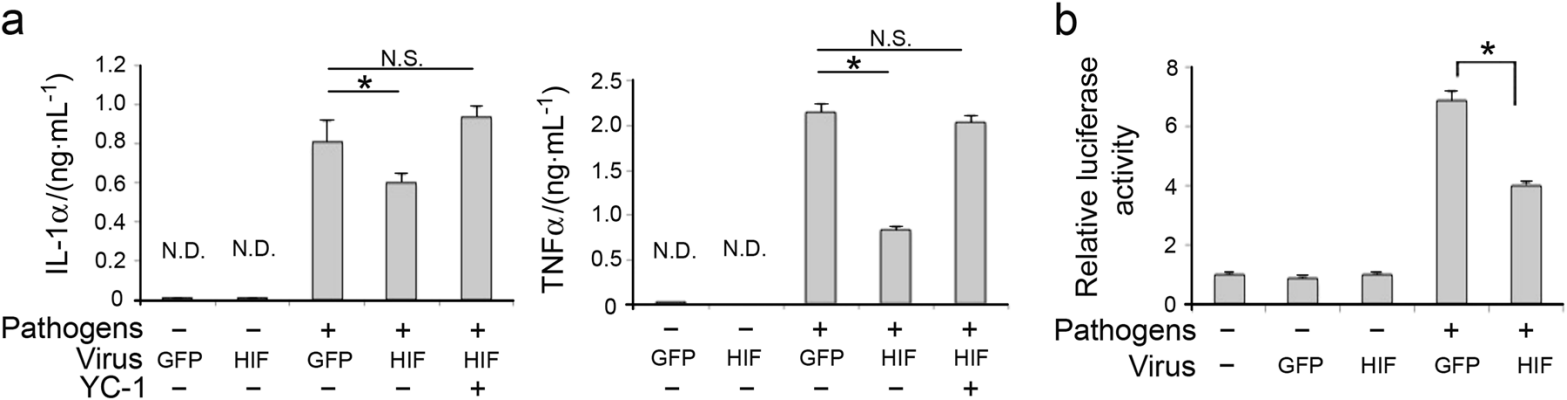
Induction of CA-HIF1A led to suppression of inflammatory response in macrophages. **a** Effect of CA-HIF1A on cytokine productions. The effect of conditions within stimulated cells was determined by one-way ANOVA with Bonferroni post hoc test, * P < 0.05 vs. Ad-EGFP, N.S., not significant, N.D., not detected, vertical bar: standard deviation, *n* = 3 per condition. **b** Effect of CA-HIF1A on NF-κB transcriptional activity. The effect of CA-HIF1A within stimulated cells was determined by Student’s *t*-test, * P < 0.05 vs. Ad-EGFP/stimulated group, vertical bar: standard deviation, *n* = 3 per condition

In addition to the pharmacological approach, we employed an adenoviral vector (Ad-) encoding CA-HIF1A to forcibly activate the HIF-1 pathway. Successful induction of CA-HIF1A and enhanced green fluorescent protein (EGFP; served as control) was preliminarily confirmed in primary macrophages (data not shown). We verified that the viral vectors alone did not cause production of proinflammatory cytokines (Fig. 4a). We then examined the effect of CA-HIF1A on the production of IL-1α and TNFα by endodontic pathogen-stimulated macrophages. Compared with EGFP controls, induction of CA-HIF1A significantly suppressed the production of IL-1α and TNFα by 75% (* P* < 0.05) and 38.8% (* P* < 0.01), respectively (Fig. 4a). Co-treatment of Ad-CA-HIF1A with YC-1 resulted in a recovery of proinflammatory cytokine productions to the levels in the positive control (stimulated/EGFP) (Fig. 4a). We also examined the effect of CA-HIF1A on NF-κB promoter activity using the reporter assay described above. As shown in Fig. 4b, the viral vectors did not solely activate NF-κB promoter activity. CA-HIF1A significantly inhibited pathogen-stimulated NF-κB promoter activity by 58.6% compared to the EGFP control.

### Local induction of CA-HIF1A attenuated development of periapical lesions

**Fig. 5:**
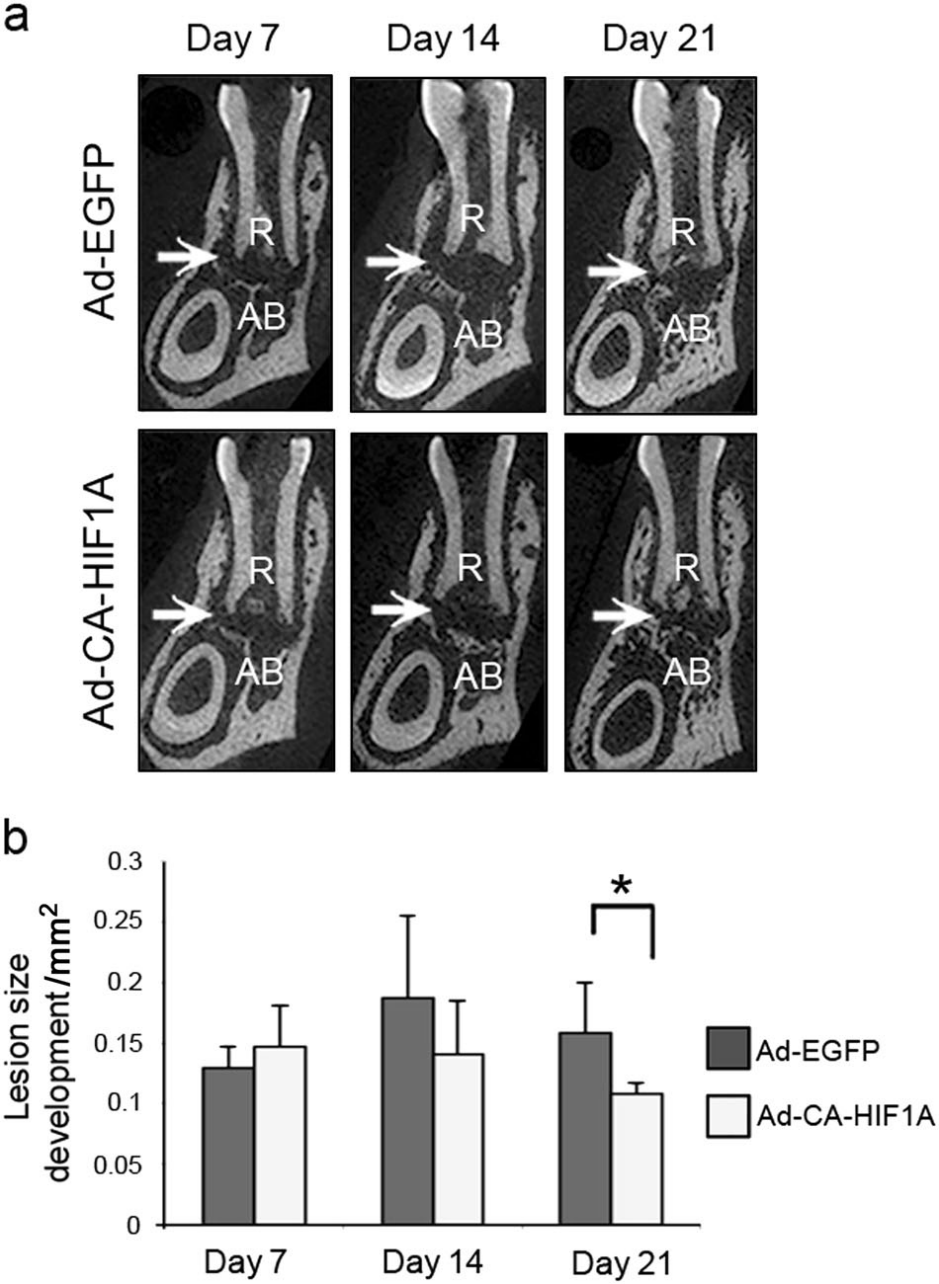
Periapical injection of Ad-CA-HIF1A attenuated development of periapical lesions. **a** Representative μCT images of periapical lesions in anterior-posterior direction. The position of each CT slice was the most central part of mandibular first molar distal root (R). Arrow points to the area of periapical lesion. The sample number was five in each group. **b** Size of periapical lesions development. The effect of treatment and observation period was determined by two-way ANOVA with Bonferroni post hoc test, *P < 0.05 vs. Ad-EGFP group, vertical bar: standard deviation, *n* = 5 per condition. *AB* alveolar bone

**Fig. 6:**
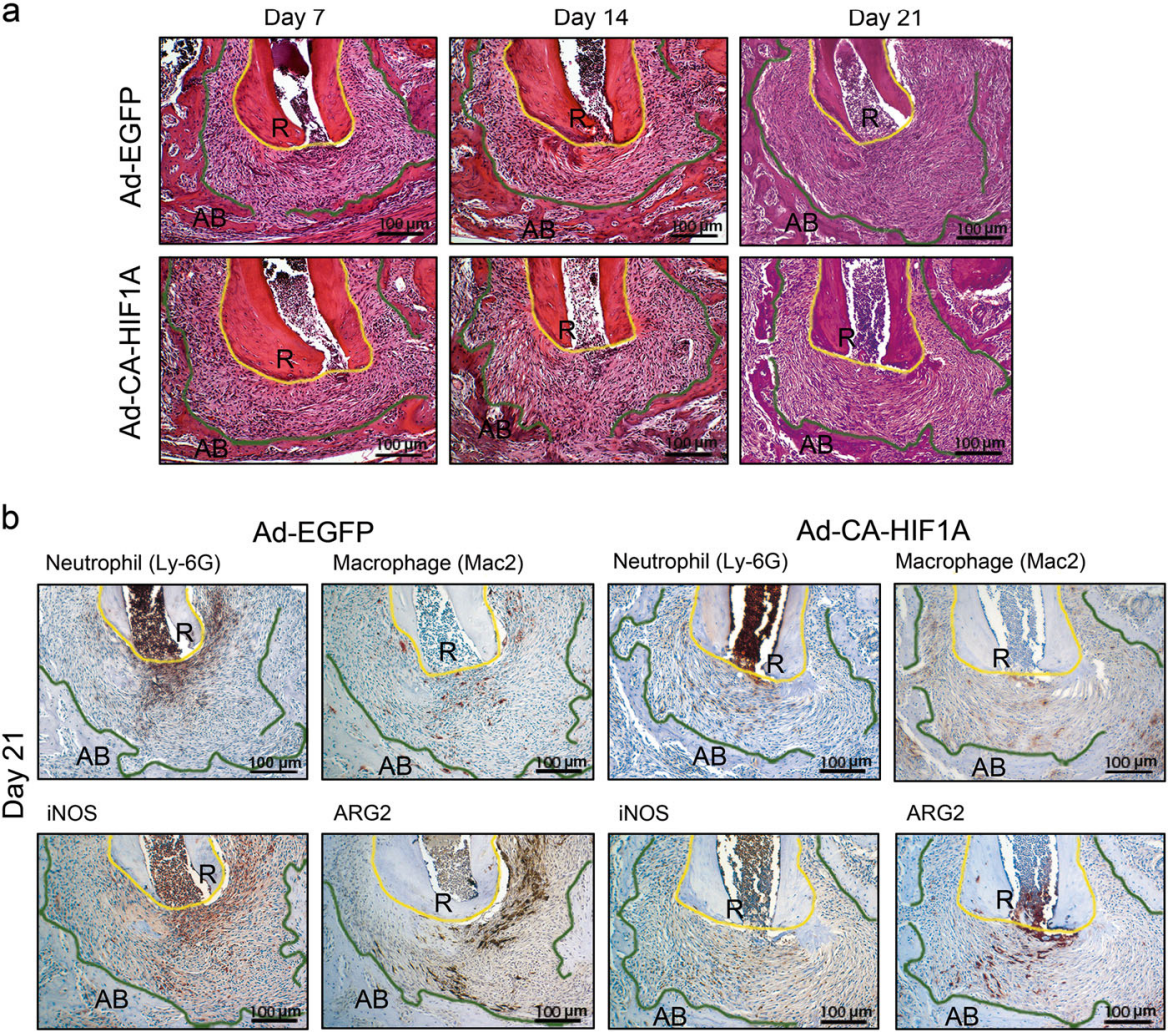
Ad-CA-HIF1A injection attenuated inflammatory cell infiltration into periapical lesions. **a** Representative histology of periapical lesions on days 7, 14 and 21 after pulp exposure (H&E staining). **b** Representative immunohistochemistry for neutrophil (Ly-6G), macrophage (Mac2), iNOS and ARG1. In both panels, polygonal yellow and green lines outline dental root (R) and the margin of residual alveolar bone (AB), respectively. The area lying between these lines is periapical lesion. The original magnification was ×100

Based on our in vitro findings above, we tested whether local activation of HIF-1 attenuates the development of periapical lesion in vivo. For this purpose, we directly injected adenoviral vectors into periapical region via dental root canals. As shown in Table 2, periapical injection of Ad-CA-HIF1A led to elevation of HIF-1α gene vs. EGFP controls on days 14 and 21. On  day 21, gene expression of proinflammatory cytokines (*Il1a, Tnf*), the NF-κB family (*Nfkb1, Rela*), *Nos2* (a proinflammatory marker) and osteoclastic *Ctsk* was significantly downregulated in Ad-CA-HIF1A-injected mice vs. Ad-EGFP-injected controls. A similar trend was observed on  day 7. However, the osteogenic genes *Runx2* and *Sp7* were slightly upregulated in earlier phases of the disease development (days 7 and 14). Another osteogenic gene *Atf4* was upregulated on  day 21. μCT analysis in the same experiment (Fig. 5) revealed that Ad-CA-HIF1A-injected animals exhibited significant suppression of periapical bone loss compared with that in Ad-EGFP-injected mice on  day 21 (32%; * P* < 0.05). Histological appearance of inflammation on  days 7 and 14 was quite similar regardless of the type of vectors used (Fig. 6a). Pulp exposure induced mild inflammatory cell infiltration in the periapical area on day 7 followed by formation of granulation tissue with moderate inflammatory cell accumulation on days 14 and 21. However, CA-HIF1A led to the development of mature granulation tissue exhibiting less inflammatory cell infiltration on day 21 compared to the EGFP group. As shown in Fig. 6b, accumulation of Ly-6G(+), Mac2(+) and iNOS(+) cells was attenuated in the CA-HIF1A group compared with EGFP controls on day 21 (Fig. 6b). Similar to the histological observations in the DMOG experiment (Fig. 2b), both iNOS(+) and ARG1(+) cells widely infiltrated the lesions of EGFP disease controls while infiltration of these cells was quite limited in the CA-HIF1A group on day 21 after pulp exposure.

**Table 2:** Real-time RT-PCR result of Ad-CA-HIF1A injection

	Day 7			Day 14			Day 21		
Gene	Ratio	CI	*P*-Value	Ratio	CI	*P*-Value	Ratio	CI	*P*-Value
*Il1a*	0.81	(0.71–0.93)	N.S.	3.12	(2.31–4.22)	0.001	0.87	(0.81–0.89)	0.03
*Tnf*	0.84	(0.80–0.88)	0.02	0.99	(1.11–0.88)	N.S.	0.33	(0.18–0.61)	0.01
*Nfkb1*	0.81	(0.68–0.96)	N.S.	1.38	(1.27–1.5)	N.S.	0.63	(0.54–0.74)	0.008
*Nfkb2*	0.58	(0.53–0.64)	0.001	1.41	(1.24–1.6)	0.020	0.36	(0.19–0.71)	N.S.
*Rela*	0.58	(0.39–0.87)	N.S.	1.36	(1.18–1.57)	N.S.	0.41	(0.23–0.71)	0.03
*Relb*	0.72	(0.59–0.87)	0.02	1.24	(1.18–1.26)	N.S.	0.88	(0.74–1.05)	N.S.
*Nos2*	1.02	(0.86–1.21)	N.S.	0.99	(0.9–1.09)	N.S.	0.45	(0.39–0.51)	0.03
*Arg1*	0.66	(0.40–1.09)	N.S.	1.95	(1.06–3.57)	N.S.	0.61	(0.32–1.15)	N.S.
*Chil3*	0.93	(0.78–1.1)	N.S.	1.08	(0.97–1.2)	N.S.	1.75	(1.19–2.57)	N.S.
*Retnla*	0.54	(0.36–0.81)	N.S.	1.13	(0.83–1.53)	N.S.	1.28	(1.02–1.62)	N.S.
*Acp5*	1.02	(0.86–1.21)	N.S.	1.31	(0.8–2.09)	N.S.	0.84	(0.65–1.07)	N.S.
*Ctsk*	0.85	(0.76–0.96)	N.S.	1.06	(0.93–1.2)	N.S.	0.49	(0.28–0.84)	0.04
*Runx2*	1.27	(1.08–1.50)	N.S.	1.30	(1.2–1.46)	N.S.	0.63	(0.45–0.89)	0.04
*Osx*	1.28	(1.11–1.48)	0.03	1.34	(1.2–1.53)	0.009	0.38	(0.23–0.62)	0.02
*Atf4*	1.24	(1.02–1.50)	N.S.	1.35	(1.21–1.51)	0.003	1.46	(1.11–1.92)	0.02
*Hif1a*	1.13	(0.98–1.30)	N.S.	1.47	(1.36–1.58)	0.001	1.90	(1.5–2.38)	0.003
*Car9*	1.04	(0.7–1.23)	N.S.	0.95	(0.88–1.02)	N.S.	1.13	(0.9–1.38)	N.S.
*Vegfa*	1.40	(1.02–1.93)	N.S.	1.37	(1.13–1.66)	N.S.	0.94	(0.7–1.23)	N.S.

Gene expression data were normalized to the internal reference gene (GAPDH) and analyzed by the 2^-ΔΔCT^ method. Ratio: the ratio of target gene expression in Ad-CA-HIF1A group relative to disease controls. The effect of treatment in each observation period was determined by Student’s* t-*test

*CI* 95% confidence interval, *N.*S. not significant (*P* ≥ 0.05)

## Discussion

In this study, we demonstrated that activation of HIF-1 exhibited an anti-inflammatory effect in periapical lesions. We utilised two approaches to activate HIF-1 under normoxia. DMOG inhibits activity of PHD, leading to stabilisation and accumulation of HIF-1α protein in the nucleus.^22^ In contrast, CA-HIF1A is a mutant protein missing an oxygen-sensing hydroxylation region (residues 392−520) and has missense mutations (Pro567Thr and Pro658Gln) compared with native HIF-1α.^23^ Thus, CA-HIF1A can independently and directly activate the HIF-1 pathway regardless of degradation of endogenous HIF-1α under normoxia.^23^ YC-1, which is an inhibitor of HIF-1, was used together with DMOG and Ad-CA-HIF1A to determine whether the effect of DMOG or Ad-CA-HIF1A is truly HIF dependent.

Both DMOG and Ad-CA-HIF1A attenuated periapical bone loss compared to the corresponding controls on day 21 after pulp exposure, indicating that activation of the HIF-1 pathway was protective in the development of periapical lesions. As the effect of DMOG and Ad-CA-HIF1A on the extent of periapical lesion was imperceptible in the early phase, the treatment seemed to suppress the progress of bone loss in the latter phase of disease development. Our histological examination revealed that infiltration of neutrophils and macrophages in DMOG-treated mice was significantly lower and quite limited in area compared with disease controls. In Ad-CA-HIF1A-injected mice, infiltration of neutrophils was notably mild and localized in the periapical area on  day 21. In contrast, control EGFP mice presented chronic active inflammation exhibiting widely extended neutrophil infiltration, which strongly overlapped with the area of iNOS(+) cells. These observations indicate that activation of HIF might relate to infiltration/resolution of myeloid cells in periapical inflammation. Furthermore, our gene expression analysis indicated a possible mechanism of the less inflammatory state mediated by activation of HIF-1α through downregulation of NF-κB activation and subsequent proinflammatory gene expressions.

In the process of periapical bone loss, macrophages are the most prominent cells in regulation of inflammatory and bone destructive lesions including production of proinflammatory/bone resorptive cytokines (IL-1 and TNFα).^25–27^ NF-κB is the central transcription factor for proinflammatory cytokines and osteoclast activity.^28^ We demonstrated that DMOG inhibited LPS-induced phosphorylation of p65 and IKK, which is essential for the NF-κB transcriptional activation.^28–30^ In addition, induction of CA-HIF1A resulted in downregulation of NF-κB promoter activity in macrophage-like RAW264.7 cells. Unexpectedly, the effect of DMOG on proinflammatory cytokines was inconsistent. Our data (Fig. 3b) demonstrated that DMOG did not decrease production of proinflammatory cytokines. However, CA-HIF1A clearly suppressed both IL-1α (−25%) and TNFα (−61%) in a HIF-dependent manner (Fig. 4a). These findings suggest that activation of the HIF-1 pathway probably suppresses the NF-κB signalling pathway and downstream cytokines in stimulated macrophages. We further showed that DMOG-activated HIF-1 led to an increased ratio of M2 macrophages to M1 cells, which may affect the course of inflammation and wound healing. Although DMOG may activate HIF-2, the effect of HIF-2 activation could be excluded using Ad-CA-HIF1A. Taken together, our in vitro data detailed the host protective mechanism mediated by activated HIF-1 at the gene expression, protein, and cellular levels.

In general, HIF-1 is considered a promoter and an enhancer of immune/inflammatory responses that maintain the host defence not only in hypoxia but also in normoxia.^3,4^ A number of articles have shown that NF-κB and HIF-1 form a positive feedback loop in activation of the inflammatory response pathway.^31–33^ HIF-1 is a direct target gene of NF-κB even under normoxia. In addition, hypoxia, which activates of HIF, promotes translocation of NF-κB subunits to the nucleus, resulting in elevation of the inflammatory response.^31^ However, recent studies have demonstrated anti-inflammatory effects of DMOG via downregulation of NF-κB activity in vitro and in vivo.^34,35^ Although both hypoxia and DMOG do stabilize HIF-1α, these previous findings showed that the functions of stabilised HIF-1α are diverse, and the functional differences appear to be in part dependent on the stabilization mechanism. More specifically, hypoxia enhances activation of NF-κB and subsequent proinflammatory cytokine production in LPS-stimulated macrophages compared with that in normoxia via the positive feedback loop described above.^31^ In contrast, DMOG exhibited an anti-inflammatory effect, including downregulation of NF-κB in vitro and in vivo, similar to a previous report that DMOG suppresses IL-1β-induced NF-κB activation in vitro and in vivo.^34^ As potential mechanisms of this anti-inflammatory effect, DMOG-mediated interference of TRAF6 signalling cascade, promotion of M2 macrophage activation and subsequent IL-10 production, reduction of inflammatory cell infiltration, and enhanced expression of protective genes, including extracellular adenosine signalling via A2B adenosine receptor (A2BAR), have been demonstrated.^12,36–39^ Our data shown in Tables 1 and 2 suggest a similar TRAF6 inhibitory mechanism in both DMOG- and CA-HIF1A-treated animals. However, both HIF-1-activating agents failed to induce M2 macrophage activation. Thus, downregulation of TRAF6 signalling seems to be the most likely mechanism of HIF-1-mediated attenuation of periapical inflammation.

Immune and skeletal systems interact with each other, share fundamental mechanisms, and therefore are involved in the pathophysiology of bone diseases including periapical lesions.^40^ In addition to immune regulation, HIF-1 plays critical roles in bone biology.^20^ Our data indicated that HIF-1 activation suppresses upstream genes of inflammatory bone loss. NF-κB is the master regulator of osteoclastogenesis and bone resorption.^28^ In the present study, both DMOG and CA-HIF1A attenuated periapical bone loss via downregulation of NF-κB and osteoclastogenesis (*Acp5, Ctsk*) compared to the corresponding controls (Tables 1 and 2). However, inhibition of IKK-NF-κB in differentiated osteoblasts maintains bone formation even in ovariectomised adult mice.^41^ In the present study, however, the effect of both DMOG and CA-HIF1A on the expression of osteogenic genes, including *Runx2, Sp7* and *Atf4* was marginal. Collectively, HIF-mediated attenuation of periapical bone loss seems to be dependent on inhibition of osteoclasts rather than promotion of osteoblasts and bone formation.

VEGF is one of the major HIF-1 target molecules and is involved in periapical pathogenesis.^9^ However, the *Vegfa* gene unexpectedly did not respond to both DMOG and CA-HIF1A in the present study (Tables 1 and 2), suggesting involvement of a HIF-independent mechanism in regulation of VEGF and angiogenesis in periapical lesions.^42^ Similarly, another downstream gene Car9 (encoding carbonic anhydrase 9) did not respond to HIF activation. The effect of activated HIF-1 on its downstream molecules is not clear. The explanation for this unresponsiveness is unknown and needs to be determined in further well-designed studies.

In this study, activation of HIF-1α was involved in host defence against periapical lesions via downregulation of NF-κB, proinflammatory/bone resorptive cytokines, M1 macrophages and osteoclastogenesis. Our findings suggest that HIF-1α might be a potential therapeutic target for apical periodontitis. However, additional studies are required to further elucidate the role of HIF in this chronic inflammation. In particular, the HIF-1/HIF-2 switch in immunomodulation, HIF-altered cell metabolism, and the molecular adaptive responses, including regulation of mitochondrial reactive oxygen species (ROS) in periapical lesions are of interest.^43,44^

## Materials and methods

### Animals

Wild-type (WT) C57BL/6J mice were purchased from The Jackson Laboratory (Bar Harbor, ME, USA). Mice were maintained in the Forsyth Institute Animal Facility (Cambridge, MA, USA) under specific pathogen-free conditions. All experimental protocols were approved by The Forsyth Institute’s Institutional Animal Care and Use Committee.

### HIF-1-activating agents and inhibitor

DMOG was purchased from Frontier Scientific (Logan, UT, USA). Recombinant adenovirus encoding CA-HIF1A (Ad-CA-HIF-1Α) was generated using the AdEasy^TM^ XL Adenoviral Vector System (Agilent, CA, USA) according to the manufacturer’s instruction. As a control, recombinant adenovirus encoding EGFP (Ad-EGFP) was employed. Viral vectors were concentrated using an Adenovirus Standard Purification ViraKit^TM^ (VIRAPUR, CA, USA), and the titer of each vector was determined by an Adeno-X^TM^ Rapid Titer Kit (TaKaRa Bio U.S.A. Inc., CA, USA). YC-1, a known HIF-1 inhibitor, was purchased from Sigma-Aldrich (St. Louis, MO, USA).

### Induction of periapical lesions and treatment regimens

Mice at 8 weeks of age were subjected to dental pulp exposure to determine the effect of DMOG in vivo.^45,46^ Briefly, mice were anesthetised with 62.5 mg·kg^-1^ ketamine HCl and 12.5  mg·kg^-1^xylazine in sterile PBS by intra-peritoneal (i.p.) injection. Mandibular first molar dental pulps were surgically exposed. The access cavity was left open to the oral cavity after removal of the pulp tissue using an endodontic file, allowing contamination of root canals with oral commensal microorganisms. Mice received i.p. injections of DMOG (1.25 mg per day) or PBS as a vehicle control from day 1 to the day before sacrifice relative to pulp exposure.^47^ Mice were sacrificed on days 10 and 21 after pulp exposure. Five-week-old mice were employed to determine the effect of CA-HIF1A in vivo. After the pulp exposure described above, either Ad-CA-HIF1A or control Ad-EGFP (10^7^ infectious units per μL PBS per tooth for one time) was injected into periapical tissue via root canals using a 36G needle mounted on a Nanofil syringe (both World Precision Instruments, Sarasota, FL, USA) set in a syringe pump (Pump 11 Nanomite; Harvard Apparatus, Holliston, MA, USA). The size of root canals at this age allows deep insertion of the 36G needle near the apical foramen. The effective dose for periapical injection was set to a dose of 1/64 the intratumour injection in 100 mm^3^ tumours, based on the total volume of periapical tissue of the mandibular first molar (≃1.6 mm^3^) estimated by μCT.^48^ Injections were carried out on days 0 (just after pulp exposure), 5, 12 and 19. Mice were euthanised on days 7 (received two injections), 14 (received three injections) and 21 (received four injections). We preliminarily confirmed that this treatment regimen resulted in successful induction of the target protein by enzyme-linked immunosorbent assay (ELISA). After euthanasia, mandibles were isolated and hemisected. One hemimandible was fixed in 4% paraformaldehyde in PBS and subjected to micro-computed tomography and histology. The other hemimandible was immediately frozen and stored in –80 °C until total RNA extraction for real-time RT-PCR.

### Micro-computed tomography

Fixed hemimandibles were scanned as previously described using a cone beam-type tomograph (μCT40, Scanco Medical, Bassersdorf, Switzerland).^24^ The size of the periapical lesions was measured based on a standardised protocol.^49^ In brief, the most centrally located section, which included the crown and distal root of the mandibular first molar and exhibited a patent root canal apex, was selected for quantitation. The cross-sectional area of distal root periapical lesions was selected and quantified using Adobe Photoshop CS6 (Adobe Systems, San Jose, CA, USA) and ImageJ (National Institutes of Health, Bethesda, MD, USA). The lesion size was obtained by subtraction of an averaged normal periodontal space in baseline controls from a total periapical radiolucent area and expressed as square millimetres.

### Histology and immunohistochemistry

After μCT, the samples were subjected to histology as previously described.^49^ In brief, fixed hemimandibles were decalcified, embedded in paraffin and sectioned at 6 μm thickness following a general histology protocol. H&E staining was used for initial histological examination. Upon the initial observation, pivotal sections containing patent root canal with localised periapical lesions were immunohistochemically stained for neutrophils (purified anti-Ly-6G; dilution 1:500; BioLegend, Inc., San Diego, CA, USA), macrophages (purified anti-Mac2 protein; dilution 1:500; BioLegend, Inc.), inducible NO synthase (iNOS) (ab3523; dilution 1:400; Abcam, Cambridge, MA, USA), and arginase 1 (ARG1) (sc-18351 (N20); dilution 1:50; Santa Cruz Biotechnology Inc., Dallas TX, USA), respectively. Primary antibodies were detected using the Liquid DAB+Substrate Chromogen System (DAKO Denmark A/S, Denmark). For enumeration of neutrophils and macrophages, a standardised guide frame was overlaid on the captured images. The target cells within the frame were counted and expressed as cells per square millimetres.

### Real-time RT-PCR

Bone blocks containing periapical lesions were isolated from hemimandibles. Total RNA extraction from bone block and following complementary DNA (cDNA) synthesis was conducted using TRIzol Reagent (Thermo Fisher Scientific, Inc. Waltham, MA, USA), Fastprep-24 with matrix A (both MP Biomedicals, Santa Ana, CA, USA), and an iScript cDNA Synthesis Kit (Bio-Rad, Hercules, CA, USA). Real-time RT-PCR was performed using a KAPA SYBR FAST qPCR Kit (KAPA Biosystems, Wilmington, MA, USA) and a Light Cycler 480 II system (Roche, Pleasanton, CA, USA). All primer sets listed in Table 3 were purchased from Real Time Primers (Elkins Park, PA, USA). The 2^−ΔΔCT^ method was used for data analyses.

**Table 3:** Primer sets used for real-time RT-PCR

Gene	Forward (5′-3′)	Reverse (5′-3′)
*Il1a*	CGGGTGACAGTATCAGCAAC	GACAAACTTCTGCCTGACGA
*Tnf*	CTATGTCTCAGCCTCTTCTC	CAGCCTTGTCCCTTGAAGAG
*Nfkb1*	TGAGAATGGACAGAACAGCA	AAGCTGAACAAACACGGAAG
*Nfkb2*	ACCTTTGCTGGAAACACACC	GTATCCCTCTCAGGCCCTTC
*Rela*	GCGGGGACTATGACTTGAAT	TCCCGTGAAATACACCTCAA
*Relb*	TGGTACTGCTAGCCTTGTGG	AGGATGAGGAAGCTGGAAGA
*Nos2*	CTTTGTGCGAAGTGTCAGTG	CACCTGGAACAGCACTCTCT
*Arg1*	GTGAAGAACCCACGGTCTGT	CTGGTTGTCAGGGGAGTGTT
*Chil3*	GGTTTTTCCACAGTGCATTC	AGCATGGTGGTTTTACAGGA
*Retnla*	TTCTTGCCAATCCAGCTAAC	GGGTTCTCCACCTCTTCATT
*Acp5*	GCAGTATCTTCAGGACGAGAAC	TCCATAGTGAAACCGCAAGTAG
*Ctsk*	CTTAGTCTTCCGCTCACAGTAG	ACTTGAACACCCACATCCTG
*Runx2*	CCAAATTTGCCTAACAGAATG	GAGGCTGTGGTTTCAAAGCA
*Sp7*	ATGGCGTCCTCTCTGCTTG	TGAAAGGTCAGCGTATGGCTT
*Atf4*	ATGGCGCTCTTCACGAAATC	ACTGGTCGAAGGGGTCATCAA
*Hif1a*	TCTGGAAGGTATGTGGCATT	AGGGTGGGCAGAACATTTAT
*Car9*	GGTGCACCTCAGTACTGCTT	TGTGGTCAGAGACCCTTCAT
*Vegfa*	AGACACACCCACCCACATAC	CAGACCACACTGAAGCCTTT
*Gapdh*	CTGGAGAAACCTGCCAAGTA	TGTTGCTGTAGCCGTATTCA

### Macrophage cultures

Resident peritoneal macrophages were isolated from WT mice (6–10 weeks of age) and seeded in 35 mm dishes (5 × 10^6^ cells per dish) or 96-well plates (10^5^ cells per well) as previously described.^45^ The cells were cultured in RPMI-1640 (Thermo Fisher Scientific) supplemented with 10% fetal bovine serum (FBS; Biowest U.S.A. Riverside, MO, USA).

For western blot analyses, the cells were treated with DMOG (100, 250 and 500  μmol·L^-1^) or DMOG (500  μmol·L^-1^)+YC-1 (10, 20 and 50  μmol·L^-1^) for 12 h under normoxia. The same volume of culture medium served a vehicle control. In some experiments, the cells were pre-treated with 500 μmol·L^-1^ of DMOG, 50 μmol·L^-1^ of YC-1 or both for 1 h. Then, the pre-treated cells were stimulated with *Escherichia coli* LPS (serotype 0111:B4; 0.1  μg·mL^-1^; Sigma-Aldrich) for 8 h.

For ELISA, the pre-treated cells were stimulated with a cocktail of common human endodontic pathogens for 24 h under the same treatment conditions. The cocktail consisted of equal number of formalin-fixed *Parvimonas micra* [American Type Culture Collection (ATCC), Manassas, VA, USA; 33270], *Streptococcus intermedius* (ATCC 27335), *Prevotella intermedia* (ATCC 25611) and *Fusobacterium nucleatum* (ATCC 25586). A total of 1 × 10^7^ cells of the fixed pathogens in 100 μL culture medium was applied to each well for stimulation. Medium alone served as a vehicle control. In some experiments, Ad-CA-HIF1A or Ad-EGFP was applied to non-treated cells [multiplicity of infection (MOI) = 1000 particles per cell] and pre-incubated for 24 h. The infected cells were pre-cultured with 50  μmol·L^-1^of YC-1 or vehicle alone for 1 h prior to the bacterial stimulation described above.

For flow cytometry analyses, the pre-treated cells were stimulated with 0.1 μg·mL^-1^of LPS for 12 h under the same treatment conditions. Non-treated/non-stimulated cells served as the baseline.

### SDS-PAGE and western blot analysis

The cells harvested from the cultures (see above) were lysed in RIPA buffer for protein extraction. The protein concentration of the extracts was quantified using Pierce^TM^ 660 nm protein assay reagent (Thermo Fisher Scientific). The extracts (25 μg of total protein per sample) were subjected to sodium dodecyl sulfate-polyacrylamide gel electrophoresis (SDS-PAGE) and blotted onto polyvinylidene fluoride (PVDF) membranes (EMD Millipore, Billerica, MA, USA) as described elsewhere.^50^ Rabbit polyclonal antibodies raised against HIF-1α (Cayman Chemical, Ann Arbor, MI, USA; dilution 1:200), total NF-κB p65, and phosphorylated NF-κB p65, phosphorylated IKK (Cell Signaling Technology, Danvers, MA, USA; 1:1000) were used in blocking buffer (5% bovine serum albumin in Tris-buffered saline). Anti-rabbit IgG conjugated with horseradish peroxidase (HRP; Cell Signaling Technology; 1:2500) was used as a secondary antibody. Beta-actin served as a loading control and was detected by anti-β-actin antibody conjugated with HRP (Abcam; 1:10,000). Target proteins on the antibody-treated membranes were visualised by an enhanced chemiluminescent method using Luminata Forte Western HRP substrate (EMD Millipore).

### ELISA

The levels of IL-1α and TNFα in the culture supernatants were determined by ELISA (DuoSets, R&D Systems, Minneapolis, MN, USA) following the manufacturer’s instructions. The concentration of target cytokine ( μg·mL^-1^ supernatant) was calculated from a standard curve that was generated from known protein standards.

### NF-κB reporter assay

The effect of DMOG or Ad-CA-HIF1A on NF-κB activity was assessed by a reporter assay using NF-κB Luciferase Stable RAW264.7 cells (NF-κB RAW; Applied Biological Materials Inc., BC, Canada). NF-κB RAW is a macrophage-like cell line stably transfected with a construct expressing firefly luciferase driven by a promoter containing an NF-κB response element. NF-κB RAW cells were seeded at 1.5 × 10^5^ cells per well in 48-well plates and pre-cultured for 6 h in Dulbecco’s modified Eagle’s medium (Thermo Fisher Scientific) supplemented with 10% FBS and G418 (100  μg·mL^-1^, Sigma-Aldrich). The cells were pre-treated with 500  μmol·L^-1^ of DMOG, 50  μmol·L^-1^ of YC-1 or both for 1 h and, then, stimulated with the endodontic pathogens (MOI = 100 bacterial cells per RAW cell) for 6 h. In another experiment, Ad-CA-HIF1A or Ad-EGFP was applied to the cells (MOI = 1000 particles per cell) and pre-incubated for 24 h prior to the bacterial stimulation described above. The luciferase activity was determined after a 6 h stimulation by the Luciferase Assay System (Promega, Madison, WI, USA) with a multimode microplate reader (Synergy HT, BioTek US, Winooski, VT, USA). The luminescence was measured for 10 s per sample.

### Flow cytometry

The effect of DMOG on differentiation of M1 and M2 macrophages from resident peritoneal macrophages was assessed by flow cytometry. After harvesting, the cells were incubated with TruStain fcX^TM^ for 10 min on ice in FACS buffer (PBS with 5% FBS and 1 mmol·L^−1^ EDTA) to block nonspecific binding of immunoglobulin to the Fc receptors, followed by incubation with fluorophore-conjugated antibodies. OneComp^TM^ eBeads (Thermo Fisher Scientific) were stained with individual fluorophore-conjugated antibodies as single-colour compensation controls. FITC-conjugated anti-CD68, APS-conjugated anti-CD80 and CP-Cy7-conjugated anti-CD206 antibodies were employed (all purchased from BioLegend) and used at a dilution of 1:100 in the FACS buffer. Note that CD68, CD80 and CD206 are considered cell surface markers for total, M1 and M2 macrophages, respectively. Expression levels of these surface markers were determined using the BD FACSAria^TM^ II cell sorter. Routinely, 50,000 cells per samples were examined. The percentage of cells staining positive for each surface protein was determined by comparing test samples with unstained and single stained samples. Data were analysed with FlowJo software (FlowJo LLC, Ashland, OR, USA).

### Statistics

Statistical analysis were performed with either one-way or two-way analysis of variance (ANOVA) and a Bonferroni post hoc test. Student’s *t*-test was used in gene expression analyses and some of the reporter assays.^51^
